# Incidence and risk factors for nursing‐related intraoperative incidents: A 5‐year retrospective study at a Japanese tertiary care hospital

**DOI:** 10.1111/jjns.70027

**Published:** 2025-09-16

**Authors:** Kazuki Mochida, Tomohiro Chaki, Michiaki Yamakage

**Affiliations:** ^1^ Department of Operating Room Nursing Sapporo Medical University Hospital Sapporo Hokkaido Japan; ^2^ Department of Anesthesiology Sapporo Medical University School of Medicine Sapporo Hokkaido Japan

**Keywords:** intraoperative incidents, nursing, patient safety, retained surgical items, specimen loss

## Abstract

**Aim:**

Nursing‐related intraoperative incidents, such as specimen loss and retained surgical items, pose serious risks to patient safety. These events may be influenced by surgical complexity, team structure, and nursing experience. However, data on the specific impact of these factors, particularly in Japan, remain limited. This study aimed to determine the incidence of nursing‐related intraoperative incidents and identify clinical and procedural factors associated with their occurrence.

**Methods:**

We conducted a retrospective observational study of all surgeries performed at a tertiary care hospital between April 1, 2018, and March 31, 2023. Data were extracted from anesthetic records, perioperative nursing notes, and an electronic incident reporting system. The primary outcome was the incidence of nursing‐related intraoperative incidents. Secondary outcomes included associations with surgical specialty, instrument delivery personnel (nurse vs. physician), duration of surgery (<2 vs. ≥2 h), timing of surgery (daytime vs. night shift), surgical approach (endoscopic vs. non‐endoscopic), and nurse experience (<6 vs. ≥6 years). Logistic regression was used for statistical analysis.

**Results:**

Of 37,265 surgical cases, 85 cases (0.23%) of nursing‐related intraoperative incidents were identified. Incident rates were highest in neurosurgery and emergency department surgery. Univariate analysis identified instrument delivery personnel (nurse), longer duration of surgery, night shift surgery, and limited nursing experience as associated factors. In multivariate analysis, limited nursing experience (odds ratio: 1.810) and longer duration of surgery (odds ratio: 4.008) remained significant predictors.

**Conclusions:**

Nursing‐related intraoperative incidents, though rare, were more likely to occur during longer surgeries and when performed by less experienced nurses.

## 1 INTRODUCTION

Ensuring patient safety in the operating room is a critical component of surgical care. Among intraoperative safety concerns, nursing‐related incidents such as specimen loss or retained surgical items can lead to serious complications, prolonged hospitalization, and legal consequences (Chen, 2025; Carmack et al., 2023). Intraoperative errors are often multifactorial, influenced by procedural complexity, communication failures, time pressure, and staffing conditions (Kolodzey et al., 2020; Santos & Johes, 2023). Nurses, particularly those responsible for instrument handling and documentation, play an essential role in maintaining intraoperative safety (Akbari et al., 2025; Singh & Arulappan, 2023). However, perioperative nurses often face stress, time pressure, and communication challenges that may compromise patient safety (Peñataro‐Pintado et al., 2021).

Retained surgical items are classified as “never events,” meaning they are considered preventable and should not occur if appropriate precautions are in place (Carmack et al., 2023). Although various safety protocols, such as surgical counts and the use of safety checklists, have been implemented to reduce these incidents, reports of retained surgical items and specimen mismanagement persist globally (Cochran, 2022; Sirihorachai et al., 2022). In particular, specialties such as neurosurgery often involve high‐complexity procedures using small, delicate instruments, making them more susceptible to item misplacement (Rigamonti et al., 2025). A recent review indicates that the successful implementation of the WHO Surgical Safety Checklist relies on effective leadership, clear communication, and staff engagement (Urban et al., 2021).

In Japan, intraoperative instrument handling may occasionally be performed by physicians instead of scrub nurses, depending on staffing conditions and institutional policies. Although limited formal data are available regarding this practice, safety concerns have been raised regarding insufficient training of non‐nursing personnel in such roles. For example, the American College of Surgeons recommends that surgical instruments be handled only by appropriately trained and credentialed staff to ensure patient safety (American College of Surgeons, n.d.). Furthermore, nurse staffing imbalances, especially during night shifts or in emergency settings, may compromise intraoperative safety. These challenges are further compounded by high nurse turnover and an increasing proportion of inexperienced staff in operating rooms, which may impact performance and increase the risk of error. Recent studies have reported that more than 70% of newly hired nurses in Japan consider leaving their positions within the first 2 years (Jiang & Kira, 2024). A recent integrative review also highlighted that turnover‐driven shortages in perioperative settings may contribute to safety risks due to the deployment of inexperienced staff (Xie et al., 2024). Although a recent nationwide study found no significant association between improved night‐shift nurse staffing and in‐hospital mortality in Japan (Morita et al., 2023), its findings underscore the complexity of evaluating nurse staffing impacts, particularly on intraoperative incidents, which remain under‐investigated.

Despite these concerns, few studies have specifically investigated the role of nursing factors, such as shift schedule, years of experience, or staffing role, in the occurrence of intraoperative incidents in Japan. Therefore, the aim of this study was to investigate the incidence of nursing‐related intraoperative incidents, including specimen loss or retained surgical items, over a five‐year period in a tertiary care hospital. We also sought to identify clinical and procedural factors associated with incident occurrence, including the experience level of the nurse in charge, the type of instrument delivery personnel, surgical approach, duration of surgery, and timing of surgery.

## 2 METHODS

### 2.1 Study design

We conducted a retrospective observational study at Sapporo Medical University Hospital, a single‐center, tertiary care institution in Japan. The study included all surgical procedures performed between April 1, 2018, and March 31, 2023, in the hospital's operating theaters. The study protocol was approved by the institutional review board (approval number: 352‐101). Written informed consent was waived due to the retrospective nature of the study. This report follows the Strengthening the Reporting of Observational Studies in Epidemiology (STROBE) guidelines.

### 2.2 Study population

All patients who underwent surgery under general, regional, or local anesthesia during the study period were considered for inclusion. In our institution, certain procedures from non‐surgical departments are also conducted in the operating theaters. For example, electroconvulsive therapy under general anesthesia for psychiatric patients is performed in the operating room. Similarly, highly invasive dermatological procedures, such as the excision of large subcutaneous tumors or multiple neurofibromas that require general anesthesia, are conducted by the dermatology department in the surgical suite. Exclusion criteria included incomplete anesthesia, surgical, or perioperative nursing records. Each surgical procedure was considered an independent unit of analysis, even if the same patient underwent multiple surgeries during the study period, as the primary focus was on surgery‐specific perioperative conditions and team configurations.

### 2.3 Data collection

Data were extracted from the hospital's electronic anesthetic record system, perioperative nursing documentation, and the institutional electronic incident reporting system. The following patient and procedural variables were collected: surgical specialty, duration of surgery, timing of surgery, urgency of procedure (elective or emergency), instrument delivery personnel (nurse or physician), surgical approach (endoscopic or non‐endoscopic) and the clinical experience of the nurse in charge (1–5 years vs. ≥6 years). In cases involving multiple surgical specialties, the procedure was attributed to the primary department responsible for the main surgical intervention.

In this study, nursing experience was defined as the number of years the nurse had been assigned to perioperative duties within the operating room. In our institution, nurses are trained to perform both scrub and circulating roles from their first year of operating room assignment, and there is no formal separation between the two roles in terms of experience tracking. Therefore, the years of experience reflect cumulative clinical exposure to both roles in the surgical setting.

For the classification of “night shift” surgeries, we defined cases as such when the patient exited the operating room during the evening or night shift periods. This definition was chosen because critical nursing tasks, such as surgical counts and specimen handling, are typically performed near the end of the procedure. Therefore, the timing of exit was considered more relevant than the start time for capturing the influence of circadian factors, fatigue, and staffing transitions on the risk of intraoperative incidents.

All nursing‐related intraoperative incidents were identified either intraoperatively during the procedure or immediately postoperatively through instrument count. All such events were documented in the incident reporting system at the time of recognition, in accordance with the institutional protocol.

### 2.4 Definition of nursing‐related intraoperative incidents

Nursing‐related intraoperative incidents were defined as adverse events involving retained surgical items (e.g., gauze, needles, instruments) or specimen mismanagement during the intraoperative phase. Only incidents confirmed through the institution's formal incident reporting system were included in the analysis. All incident reports submitted through the institutional electronic reporting system were initially reviewed and classified by one anesthesiologist and one operating room nurse in consensus. Based on this review, nursing‐related intraoperative incidents were identified for further analysis. To support accurate reporting, the institution provides annual risk management training to all operating room staff, emphasizing the importance of timely and precise documentation of intraoperative safety events.

### 2.5 Study outcomes

The primary outcome of this study was the incidence of nursing‐related intraoperative incidents, including specimen loss and retained foreign bodies. The secondary outcomes were the incidences of the nursing‐related intraoperative incidents by surgical specialty, job type of instrument delivery worker (operating nurse or doctor), duration of surgery (<2 vs. ≥2 h), timing of surgery (daytime or night shift), surgical approach (endoscopic or non‐endoscopic) and length of service of the nurse in charge (1–5 or ≥6 years).

### 2.6 Statistical analysis

Given the retrospective design, no a priori sample size calculation was performed. However, we conducted a supplementary power assessment to evaluate whether the available sample size was sufficient to detect clinically meaningful associations. Based on the observed incident rate and distribution of nurse experience, the dataset was determined to offer adequate power to identify moderate effect sizes, such as an odds ratio of 2.0, which is considered clinically relevant according to prior studies (G*power 3.1, Heinrich‐Heine‐University, Düsseldorf, Germany) (Moffatt‐Bruce et al., 2014).

Categorical variables were presented as frequencies and percentages. Univariable logistic regression was performed to identify potential predictors of incident occurrence. Variables with a *p*‐value <.05 in univariate analysis were included in multivariate logistic regression models to determine independent associations. To assess multicollinearity among the independent variables, variance inflation factors (VIFs) were calculated. Odds ratios with 95% confidence intervals were calculated. A *p*‐value less than .05 was considered statistically significant. Statistical analysis was performed using SPSS (IBM SPSS Statistics for Windows, version 25, IBM Corporation, NY).

## 3 RESULTS

### 3.1 Incidence of nursing‐related intraoperative incidents

**FIGURE 1:**
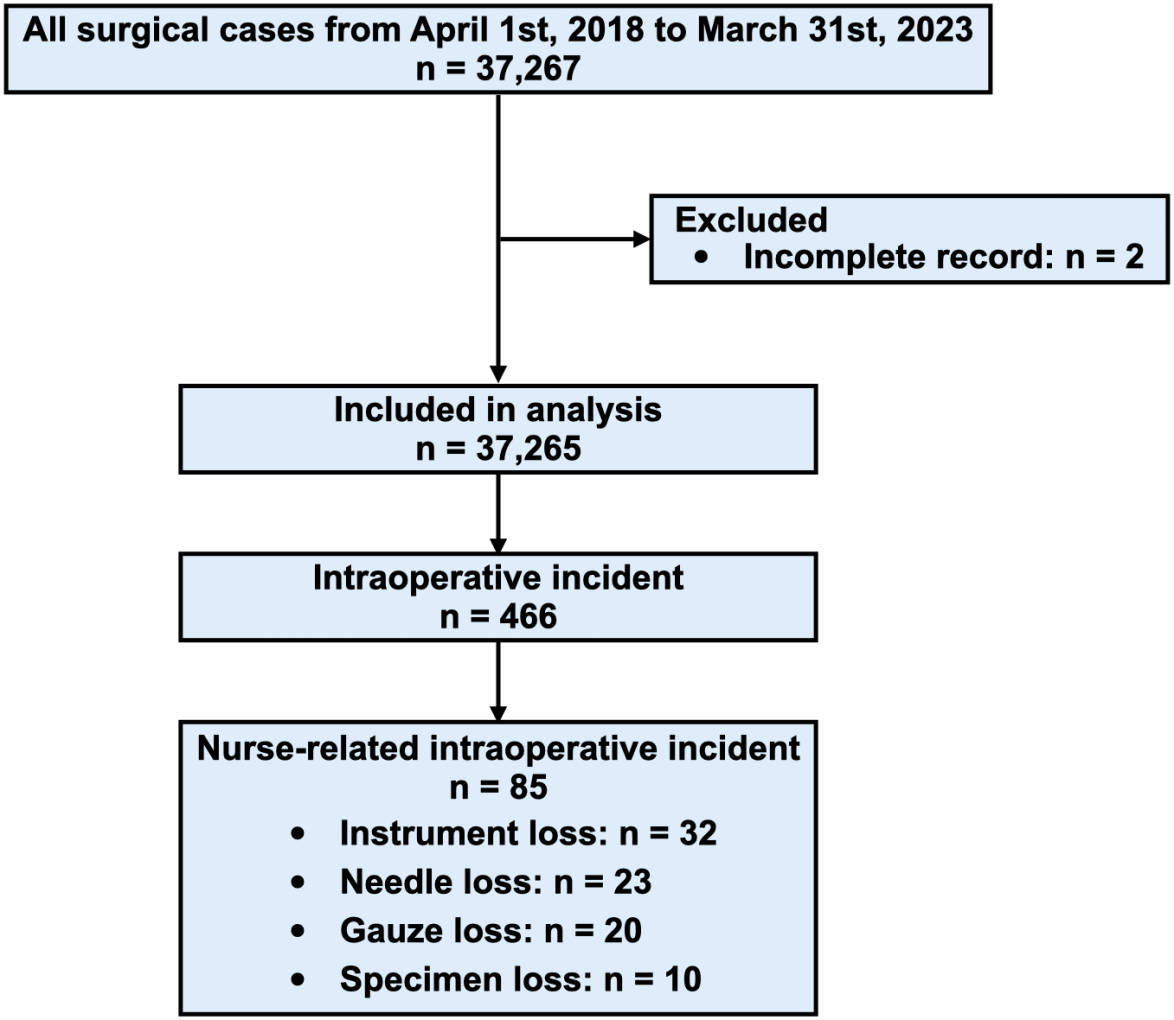
Flow diagram of surgical cases, exclusions, and incidence of intraoperative events, including nursing‐related cases.

A total of 37,267 surgical cases were extracted from April 1, 2018, to March 31, 2023, and 2 cases were excluded from the analysis due to incomplete records. 466 (1.25%) incident reports were extracted. Among them, 85 cases (0.23%) of specimen loss or retained foreign body occurred. In detail, there were 32 cases (0.09%) of instrument loss, 23 cases (0.06%) of needle loss, 20 cases (0.05%) of gauze loss, and 10 cases (0.03%) of specimen loss (Figure 1). The incidence of nursing‐related intraoperative incidents was frequent in the emergency department surgery (4/367, 1.09%), neurosurgery (10/1209, 0.83%), orthopedics (16/5008, 0.32%), cardiovascular surgery (4/1453, 0.28%), and oral surgery (6/2387, 0.25%). Notably, four emergency surgeries were neurosurgical procedures, suggesting that neurosurgery represents the highest‐risk category. The comprehensive rates of nursing‐related intraoperative incidents are presented in Table 1.

**TABLE 1:** Occurrences of nurse‐related intraoperative incidence.

Department	Occurrence of nurse‐related‐intraoperative incident (%)	Gauze	Needle	Surgical equipment	Specimen
Emergency	1.090 (4/367)	0	3	1	0
Neurosurgery	0.827 (10/1209)	6	1	2	1
Gastrointestinal surgery	0.463 (19/4101)	7	1	10	1
Orthopedics	0.319 (16/5008)	0	6	8	2
Cardiovascular surgery	0.275 (4/1453)	0	3	1	0
Oral surgery	0.251 (6/2387)	3	1	2	0
Dermatology	0.202 (3/1484)	1	0	1	1
Urology	0.159 (4/2512)	1	0	2	1
Ophthalmology	0.113 (6/5291)	0	6	1	0
Obstetrics and Gynecology	0.112 (5/4456)	0	0	2	3
Otolaryngology	0.105 (3/2850)	1	1	0	1
Plastic surgery	0.098 (4/4085)	0	2	2	0
Thoracic surgery	0.069 (1/1443)	1	0	0	0
Psychology	0 (0/534)	0	0	0	0
Total	0.232 (85/37,265)	20	23	32	10

### 3.2 Factors influencing the occurrence of nursing‐related intraoperative incidents

VIFs for all independent variables ranged from 1.00 to 1.25, confirming the absence of significant multicollinearity. Univariate analysis was performed using medical specialty, job type of instrument delivery worker, duration of surgery, timing of surgery, surgical approach, and length of service of the nurse in charge to identify the possible factors influencing the occurrence of nursing‐related intraoperative incidents. It suggested that the following factors were significantly associated with nursing‐related intraoperative incidents: instrument passing during surgery by a nurse (odds ratio: 1.567, 95% confidence interval: 1.011–2.433, *p* = .049), longer duration of surgery (≥2 h, odds ratio: 4.415, 95% confidence interval: 2.783–7.114, *p* < .001), night shift (odds ratio: 2.185, 95% confidence interval: 1.421–3.416, *p* < .001), and nurses who had been practicing for fewer than 6 years (odds ratio: 1.784, 95% confidence interval: 1.163–2.769, *p* < .001) (Table 2). In contrast, the urgency of surgery (elective vs. emergency) and surgical approach (endoscopic vs. non‐endoscopic) were not significantly associated with incident risk in univariate analysis and were therefore not included in the multivariate model.

**TABLE 2:** Univariate logistic regression analysis of the factors for nurse‐related intraoperative incidence.

Variables	Incident (+)	Incident (−)	Odds ratio	95% CI	*p* value
Instrument delivery personnel					
Physician	31	17,608			
Nurse	54	19,572	1.567	1.011–2.433	.049
Duration of surgery					
<2 h	23	23,086			
≥2 h	62	14,094	4.415	2.783–7.114	<.001
Timing of surgery					
Day time	34	22,047			
Night shift	51	15,133	2.185	1.421–3.416	<.001
Urgency of surgery					
Elective surgery	78	34,180			
Emergency surgery	7	3000	1.022	0.469–2.140	.843
Experience level of assigned nurse					
≥6 years	35	20,649			
1–5 years	50	16,531	1.784	1.163–2.769	<.001
Surgical approach					
Endoscopic	24	8189			
Non‐endoscopic	61	28,991	0.718	0.445–1.136	.189

Abbreviation: CI, confidence interval.

Based on the results of univariate analysis, multivariate analysis was performed to identify the significant factors influencing the occurrence of nursing‐related intraoperative incidents. The multivariate analysis revealed that nurses who had been practicing for fewer than 6 years (odds ratio: 1.810, 95% confidence interval: 1.178–2.812, *p* = .007) and longer durations of surgery (odds ratio: 4.008, 95% confidence interval: 2.398–6.903, *p* < .001) were significantly related to the occurrence of nursing‐related intraoperative incidents (Table 3).

**TABLE 3:** Multivariate logistic regression analysis of the factors for nurse‐related intraoperative incidence.

Variables	Odds ratio	95% CI	*p* value
Instrument delivery personnel (nurse)	1.008	0.621–1.609	.975
Duration of surgery (≥2 h)	4.008	2.398–6.903	<.001
Timing of surgery (night shift)	1.521	0.964–2.427	.067
Experience level of assigned nurse (1–5 years)	1.810	1.178–2.812	.007

Abbreviation: CI, confidence interval.

## 4 DISCUSSION

This retrospective study investigated the incidence and associated factors of nursing‐related intraoperative incidents, such as specimen loss and retained foreign bodies, over a 5‐year period at a tertiary care institution. Among 37,265 surgical cases, 466 intraoperative incident reports (1.25%) were identified, of which 85 cases (0.23%) involved specimen loss or retained surgical items. Neurosurgery and emergency department surgery showed a higher frequency of such incidents. Multivariable analysis revealed that nurses with fewer than 6 years of clinical experience and more than 2 h of surgery duration were significantly associated with increased incident risk.

### 4.1 Incidence of nursing‐related intraoperative incidents

The increased incidence of intraoperative incidents in neurosurgical procedures may be attributed to the use of small, intricate materials, such as micro sutures and tiny gauze pieces, which are more susceptible to misplacement or loss (Rigamonti et al., 2025; Weprin et al., 2021). Similarly, the higher occurrence of incidents in emergency department surgeries, particularly those involving neurosurgical procedures, may be due to both the complexity of the procedures and environmental factors, such as insufficient staffing during urgent situations. In addition to neurosurgery and emergency surgery, other specialties such as orthopedics and gastrointestinal surgery also demonstrated relatively elevated rates of nursing‐related intraoperative incidents. In orthopedic procedures, the frequent use of gauze, metallic implants, and specialized surgical instruments may increase the risk of item loss. This is particularly true for complex trauma or spinal surgeries that require extensive instrumentation. For gastrointestinal surgery, surgical equipment loss was the predominant issue. This may be due to the use of numerous endoscopic tools, energy devices, and detachable accessories, which are sometimes difficult to track visually or manually during long or technically demanding procedures. These specialty‐specific characteristics likely contribute to increased intraoperative complexity and handling burden for perioperative staff, thereby raising the potential for nursing‐related errors. To mitigate these risks, it is essential to enhance staffing levels and establish a robust on‐call system, especially for emergency department surgeries and neurosurgical procedures. The prevalence of retained surgical items may also be linked to the increasing complexity of procedures, such as robotic and implant surgeries, which involve specialized instruments (Hempel et al., 2015). In addition, a meta‐analysis identified multiple intraoperative conditions, such as prolonged duration of surgery, multiple surgical teams, and lack of formal counts, as significant contributors to retained items (Moffatt‐Bruce et al., 2014). Our institution utilizes four different types of surgical robots, each requiring distinct sets of instruments, necessitating meticulous coordination with sterilization departments and equipment manufacturers to ensure proper instrument management.

### 4.2 Role of instrument delivery personnel

Nurses with specialized training in instrument handling may reduce the risk of retained surgical items compared to physicians performing the same tasks (Stawicki et al., 2013). However, our study did not find a significant difference in incident rates based on whether a nurse or a physician was responsible for delivering the instrument. Notably, at our institution, physicians perform instrument delivery in approximately 47.4% of surgeries, a rate nearly double the national average of 25.2% reported by the Japanese Association of Operating Room Nurses in 2021, due to the limited availability of nurses. This higher rate may indicate that physicians at our institution are more accustomed to instrument delivery, which could potentially explain the lack of difference in incident rates. Nevertheless, these findings are specific to our institution and may not be applicable to other institutions.

### 4.3 Duration of surgery

Our analysis revealed that surgeries lasting over 2 h had an approximately fourfold increase in incident rates. At our institution, about 37% of surgeries exceed this duration. To reduce the occurrence of incidents, it is advisable to assign more experienced nurses to longer surgeries. This approach aligns with previous findings demonstrating that increased mental fatigue among operating room nurses is significantly associated with higher rates of missed perioperative care (Rahmani et al., 2025). In addition to staffing considerations, introducing structured fatigue mitigation strategies, such as scheduled intraoperative breaks or team rotation protocols, may also help maintain vigilance during prolonged procedures (Booker et al., 2024). Recent evidence suggests that sufficient rest periods during extended shifts contribute to sustained alertness and reduced fatigue‐related errors among healthcare professionals (Watanabe et al., 2025).

### 4.4 Timing of surgery

While univariate analysis indicated a higher incidence of intraoperative incidents during night shifts, this factor was not significant in multivariate analysis. Night shifts are known to disrupt circadian rhythms, leading to increased fatigue and decreased vigilance among healthcare workers (Moosavi et al., 2025). However, in our study, the lack of statistical significance in the multivariate model may be explained by several factors. One possibility is confounding by nurse experience or surgical complexity, both of which may have overlapped with nighttime procedures. In our institution, more experienced nurses are often assigned to cover night shifts, particularly for high‐risk or emergency procedures. This allocation strategy may mitigate the increased risk typically associated with night work, thus reducing the apparent independent effect of shift timing on incident occurrence.

Furthermore, although night‐time surgeries often include urgent cases, they may also involve shorter or less complex procedures compared to daytime elective surgeries. These factors could contribute to a dilution of the association between night shift and incident risk after adjustment for other covariates. Another explanation could be the relatively smaller number of surgical cases conducted during night shifts, which may have reduced the statistical power to detect an independent association.

### 4.5 Experience level of assigned nurse

Our findings indicate that assigning nurses with six or more years of experience to surgeries is associated with a significant reduction in incident rates. However, our institution faces challenges with nurse turnover, resulting in a higher proportion of nurses with less than 6 years of experience. While an international study suggested that a higher staffing ratio may contribute to improved patient safety (Twigg et al., 2011), such benchmarks reflect conditions in high‐resource settings like Australia. In contrast, Japanese hospitals typically operate under stricter human resource constraints. For example, under the Japanese medical fee reimbursement system, the basic staffing standard for operating rooms requires two nurses per case (one scrub nurse and one circulating nurse), and any additional support must be covered within limited institutional resources (Ministry of Health, Labour and Welfare, 2022). Given these constraints, direct application of international benchmarks must be contextualized. Therefore, safety strategies should consider local workforce realities and aim to optimize existing staffing models rather than rely solely on increasing headcount. Addressing this shortfall through targeted recruitment and retention strategies, as well as enhancing training programs for less experienced nurses, is crucial to improving patient safety outcomes (Aiken et al., 2014). In addition to improving staffing balance, the implementation of structured simulation‐based training programs may serve as a practical and effective approach to bridge the experience gap. Such programs can help less experienced nurses gain confidence and competence in high‐risk perioperative scenarios, thereby reinforcing safety practices and reducing the likelihood of intraoperative errors (Lewis et al., 2019).

### 4.6 Limitation

This study has several limitations. First, it was conducted at a single tertiary care hospital, which may limit the generalizability of the findings to other institutions with different staffing structures and workflows. Second, the retrospective design relied on incident reporting systems, which are subject to underreporting and variability in documentation accuracy. Third, we did not assess the level of intraoperative complexity in detail, which could have influenced the incidence of nursing‐related incidents. In addition, data on the experience levels of surgical team members, including both primary surgeons and physicians who served as instrument delivery personnel, were not systematically recorded in the institutional records. This limitation prevented us from evaluating whether the clinical experience of these individuals influenced the risk of nursing‐related intraoperative incidents. While our analysis included nursing experience and personnel roles, these other staffing factors should be addressed in future prospective studies. Additionally, some procedures involved collaboration among multiple specialties; in such cases, we categorized the case based on the primary surgical specialty responsible for the main procedure. In addition, the same patient may have undergone multiple surgeries during the study period; however, each procedure was treated as an independent observation. While this approach reflects real‐world surgical episodes and perioperative team configurations, it may introduce potential clustering effects that were not accounted for in our statistical models. Furthermore, due to the limited number of incidents within most surgical specialties, we were unable to perform subgroup analyses to determine whether the association between nurse inexperience and incident occurrence varied by specialty or procedure type. Such analyses would have lacked statistical power and may have produced unstable estimates. Lastly, potential confounding factors such as team communication, operating room dynamics, and nurse fatigue levels were not evaluated, all of which may contribute to intraoperative risk. In particular, latent team‐based elements such as intraoperative surgeon‐nurse communication quality, overall teamwork climate, and operating room culture may have played a role in incident occurrence but are difficult to quantify retrospectively. Future prospective studies should incorporate these variables, along with standardized assessments of workload and fatigue, and should consider multicenter designs that enable more robust stratified analyses across surgical specialties. This would allow for a more comprehensive understanding of the multifactorial causes of intraoperative nursing‐related incidents and help guide the development of targeted preventive strategies.

## 5 CONCLUSION

This retrospective study identified that nursing‐related intraoperative incidents occurred in 0.23% of surgeries, with a higher frequency in neurosurgery and emergency department surgery. The most significant factors associated with incident occurrence were a longer duration of surgery and nursing experience, particularly among those with less than 6 years of practice. Improving nurse staffing, especially with experienced personnel, and enhancing training and support systems are essential to reducing incident risk. Institutional efforts to prioritize experienced staffing and structured training for high‐risk surgeries are warranted.

## AUTHOR CONTRIBUTIONS

Kazuki Mochida and Tomohiro Chaki contributed to all aspects of this manuscript, including study conception and design, analysis and interpretation of data, and drafting the article. Michiaki Yamakage contributed to study conception and design, interpretation of data, and revising the article critically for important intellectual content.

## CONFLICT OF INTEREST STATEMENT

The authors declare that there is no conflict of interest.
